# Evaluation of the absolute oral bioavailability of the anaplastic lymphoma kinase/c-ROS oncogene 1 kinase inhibitor lorlatinib in healthy participants

**DOI:** 10.1007/s00280-021-04368-1

**Published:** 2021-10-26

**Authors:** Jennifer E. Hibma, Melissa O’Gorman, Sunil Nepal, Sylvester Pawlak, Katherine Ginman, Yazdi K. Pithavala

**Affiliations:** 1 Pfizer (Clinical Pharmacology Oncology), La Jolla, 10555 Science Center Drive, CB10-2727, San Diego, CA 92121 USA; 2 Pfizer (Pharmacometrics Oncology), Groton, CT USA; 3 Pfizer (Statistics Oncology), Groton, CT USA; 4 Pfizer (Clinical Development and Operations), New Haven, CT USA; 5 Pfizer (Clinical Development and Operations), South Lyon, MI USA; 6 Independent Statistical Consultant, Schwenksville, PA USA

**Keywords:** Absolute bioavailability, Lorlatinib, Absorption, Pharmacokinetics

## Abstract

**Purpose:**

Lorlatinib is a third-generation tyrosine kinase inhibitor currently approved for the treatment of anaplastic lymphoma kinase (ALK)-positive metastatic non-small cell lung cancer. This open-label, phase 1, randomized two-sequence, two-treatment, two-period, crossover study investigated the absolute oral bioavailability of lorlatinib in healthy participants.

**Methods:**

Eligible participants were randomized to receive two treatments in one of two sequences: lorlatinib 100 mg single oral dose followed by lorlatinib 50 mg intravenous (IV) dose, or lorlatinib IV dose followed by lorlatinib oral dose, each with at least a 10-day washout between successive lorlatinib doses. Blood samples for pharmacokinetics were collected for up to 144 hours (h) after dosing. Validated liquid chromatographic-tandem mass spectrometry was used to determine plasma concentrations of lorlatinib and its benzoic acid metabolite PF-06895751.

**Results:**

In total, 11 participants were enrolled (mean age 37.6 years, all male). The adjusted geometric mean (90% confidence interval) for the absolute oral bioavailability was 80.78% (75.73–86.16%). Using non-compartmental analysis, the estimated arithmetic mean elimination plasma half-life of lorlatinib was 25.5 and 27.0 h after the oral and IV doses, respectively. No deaths, serious adverse events (AEs), or severe AEs were reported, and most treatment-emergent AEs were mild in severity, with two events of transaminase increase of moderate severity. All treatment-emergent AEs were resolved by the end of the study.

**Conclusion:**

Both oral and IV lorlatinib were well-tolerated in healthy participants and oral lorlatinib is highly bioavailable after oral administration.

## Introduction

Lorlatinib (Lorbrena; Lorviqua EU) is a potent, orally available, brain-penetrating, third-generation anaplastic lymphoma kinase (ALK)/c-ROS oncogene 1 (ROS1) tyrosine kinase inhibitor (TKI) with a broad spectrum of clinical activity against multiple ALK resistance mutations [1]. In the phase 1/2 clinical study B7461001 (NCT01970865), lorlatinib demonstrated clinically meaningful benefit in patients with advanced non-small cell lung cancer (NSCLC), including those with intracranial metastases after progression on second-generation ALK inhibitors, and also had a favourable safety and tolerability profile [2, 3]. The activity of lorlatinib was confirmed in a large, randomized, phase 3 study B7461006 (CROWN; NCT03052608) testing lorlatinib versus crizotinib in patients with previously untreated ALK-positive advanced NSCLC. Currently, lorlatinib is approved at a 100 mg daily oral dose in the US, EU Japan, and many other countries for the treatment of adults with metastatic ALK-positive NSCLC [4, 5].

Lorlatinib is rapidly absorbed with peak plasma concentrations (C_max_) achieved 0.5–4 hours (h) after a single 100 mg oral dose [6]. Lorlatinib has demonstrated dose-linearity after single-dose administration in the range of 10–200 mg [7]. The mean (CV%) plasma terminal half-life (t½) of lorlatinib is approximately 24 h (40%) [6]. Lorlatinib is extensively metabolized, mainly by cytochrome P450 (CYP) 3A-mediated oxidation (N-demethylation, N-oxidation) and N-glucuronidation via uridine diphosphate-glucuronosyltransferase 1A4, with minor contributions from CYP2C19, CYP2C8, and CYP3A5 [6]. Lorlatinib demonstrates time-dependent pharmacokinetics (PK) resulting from simultaneous autoinhibition and auto-induction of CYP3A. The mean (CV%) oral apparent clearance (CL/F) after a 100 mg single oral dose is 11 L/h (35%) which increases to 18 L/h (39%) at steady-state, indicating net auto-induction [6]. The plasma PK of lorlatinib is not affected in a clinically meaningful manner by food or the co-administration of the proton pump inhibitor, rabeprazole [6]. However, lorlatinib plasma exposure can be affected by inducers or inhibitors of CYP3A [8–10].

Drugs with low absolute oral bioavailability are more prone to having high inter-subject variability in pharmacokinetics, which is a deterrent to providing consistent plasma exposures in the treatment population. This is one reason for clinically determining the oral bioavailability of drugs. The conduct of a crossover absolute availability study also makes it possible to directly compare the inter-subject variability of the drug clearance after oral and intravenous (IV) dosing in the same group of subjects; this allows for the evaluation of whether the variability in pharmacokinetics for the drug is primarily from metabolism or absorption.

In a previously conducted mass balance study, the percentage of unchanged lorlatinib recovered in urine and faeces following a 100 mg single oral dose of radiolabelled [14C]lorlatinib in healthy participants was < 1 and ~ 9%, respectively. The total drug-related radioactivity (parent plus metabolites) recovered in urine was 48 and 28% depending on radioactivity placement on the molecule. The most abundant circulating lorlatinib metabolite in humans is the benzoic acid metabolite, PF-06895751, formed from the intramolecular cleavage of the ring structure of lorlatinib. Based on the results from in vitro potency assays against ALK and ROS1, PF-06895751 is considered pharmacologically inactive. This metabolite accounted for 21% of the circulating drug-related radioactivity in plasma in the mass balance study [11].

Although the PK of lorlatinib has been investigated extensively, both preclinically and clinically in patients with NSCLC and in healthy participants, the absolute bioavailability of lorlatinib had not been previously characterized. As noted earlier, if the inter-subject variability in pharmacokinetics is substantial after oral dosing compared to IV dosing, this suggests that the lion’s share of the variability is from the absorption of the drug compared to metabolism and will drive the need for perhaps developing a better oral formulation. This study was hence designed to evaluate the absolute bioavailability of a single oral dose of lorlatinib relative to an IV dose administered to healthy participants. The secondary objective was to assess the safety and tolerability of both formulations in healthy participants.

## Materials and methods

### Study design

This was an open-label, phase 1, randomized two-sequence, two-treatment, two-period, crossover study in healthy participants. Approximately 12 participants were to be randomized in a 1:1 ratio to receive one of two treatment sequences: lorlatinib 50 mg IV dose (Treatment A) followed by a lorlatinib 100 mg single oral dose (Treatment B), or lorlatinib oral dose followed by lorlatinib IV dose. The IV dose is 100% bioavailable and was, therefore, considered to be the reference in this study. The reference lorlatinib 50 mg single IV dose was administered as 250 mL IV solution (0.20 mg/mL) over approximately 1.25 h at a constant rate in the morning of Day 1. Lorlatinib 100 mg single oral dose was administered as 4 × 25 mg immediate-release free base tablets in the morning of Day 1 with 240 mL ambient temperature water. Participants were instructed to swallow the lorlatinib tablets whole and to not manipulate or chew the tablets prior to swallowing. Both treatments were administered after an overnight fast of at least 10 h. To standardise the conditions on PK sampling days, during the first 4 h after dosing or start of the IV dose, participants were to refrain from eating and drinking beverages other than water or lying down (unless required for safety monitoring). Period 1 was defined as a 7-day period in which participants received Treatment A or B. Period 2 was defined as the 7-day period after Period 1, in which participants received the alternative treatment to before. Following treatment administration, participants were to undergo PK sampling for 144 h with a washout period of at least 10 days between successive lorlatinib doses. The study was conducted at a single Pfizer Clinical Research Unit (CRU) in New Haven, Connecticut, and participants were confined to the CRU from the time of admission (Day 0) until discharge (Day 7 of Period 2).

### Justification for doses used in the study

The recommended phase 2 dose of lorlatinib was determined to be 100 mg administered orally once daily based on the totality of safety, efficacy, and clinical pharmacology data [12]. Lorlatinib has been well-tolerated in clinical studies following multiple dosing in patients with NSCLC (single doses up to 200 mg) and in healthy volunteers (up to 100 mg) [2, 3, 8–12]. Hence, for the oral treatment, a 100 mg single oral dose in healthy participants was utilised in this study.

Prior to the conduct of this clinical study, lorlatinib had not previously been administered by the IV route to humans. As part of the original IND submission for oral use in humans, a battery of IV dosing toxicology studies had already been conducted in two animal species, and hence the safety of the drug following IV dosing had been appropriately characterized to meet regulatory requirements prior to initiating this study. Toxicology studies conducted with the planned IV dose formulation did not indicate any potential to cause hemolysis in human blood in vitro and studies to assess local irritation did not indicate any local vascular tissue irritation in rabbits.

Assuming a conservative absolute bioavailability of 50%, a 50 mg IV dose was predicted to produce a C_max_ approximately equivalent to the C_max_ of a 100 mg oral dose and comparable systemic exposure. The oral bioavailability of lorlatinib was anticipated to be moderate to high (≥ 48%) based on data from the mass balance study in humans described above, and from animal studies; the absolute bioavailability of lorlatinib was approximately 100% in rats and 97% in dogs. Although the absolute bioavailability of lorlatinib was unknown, a 50 mg IV dose was considered an appropriate dose to ensure healthy volunteer safety and to make sure that unusually high exposures were not generated in case the bioavailability was unexpectedly low.

### Participants

Screening procedures were performed within 28 days prior to administration of lorlatinib (Day 1) in Period 1. Eligible participants were to be healthy male or healthy female participants of non-childbearing potential. At the time of screening, participants were to be between the ages of 18 and 55 years old, with a body mass index (BMI) of 17.5–30.5 kg/m^2^, and a total body weight > 50 kg. Participants were required to be in good health based on medical history and full physical examination including vital signs, 12-lead ECG, and clinical laboratory tests. Male participants able to father children were required to refrain from sperm donation and to use a highly effective method of contraception, i.e., one that results in a failure rate of less than 1% per year when used consistently and correctly, for the duration of the study and for at least 90 days after the last dose of lorlatinib.

Participants were excluded from the study if they had evidence or a history of clinically significant haematological, renal, endocrine, pulmonary, gastrointestinal, cardiovascular, hepatic, psychiatric, neurologic, allergic disease, ongoing cardiac dysrhythmias of National Cancer Institute (NCI) Common Terminology Criteria for Adverse Events (CTCAE) Grade ≥ 2, uncontrolled atrial fibrillation of any grade, bradycardia, congenital long QT syndrome, or any condition that could possibly affect drug absorption (e.g., gastrectomy). Participants were also excluded if they had a history of regular alcohol consumption exceeding 7 drinks/week for females or 14 drinks/week for males within 6 months of screening. Additional exclusion criteria included participants with supine BP ≥ 140 mm of mercury (mm Hg) (systolic) or ≥ 90 mm Hg (diastolic) following at least 5 min of supine rest, a supine 12-lead ECG demonstrating corrected QT wave interval > 450 ms, a PR interval > 180 ms or a QRS interval > 120 ms, aspartate aminotransferase (AST)/serum glutamic oxaloacetic transaminase or alanine aminotransferase (ALT)/serum glutamic pyruvic transaminase (SGPT) ≥ 1.5 × upper limit of normal (ULN), and total bilirubin ≥ 1.5 × ULN. Participants with a positive urine test for illicit drugs, with a positive HIV or hepatitis B or C test, with current use of prescription or non-prescription drugs and/or dietary supplements (excluding paracetamol ≤ 1 g/day) within 7 days (or 5 half-lives, whichever was longer), herbal supplements and hormone replacement therapy within 28 days, treatment with an investigational drug within 30 days (or 5 half-lives, whichever was longer), and previous treatment with lorlatinib, were also excluded. Limited use of non-prescription medications that were not believed to affect participant safety or the overall results of the study were permitted on a case-by-case basis. Female participants who were pregnant or breastfeeding, and male participants with partners currently pregnant were excluded from the study. Participants could withdraw from the study at any time or could be withdrawn at any time at the discretion of the investigator or sponsor for safety, behavioural or administrative reasons.

This study was conducted in compliance with the ethical principles originating in or derived from the Declaration of Helsinki and in compliance with all International Council for Harmonisation Good Clinical Practice Guidelines. The final study protocol and informed consent documentation were approved by the Institutional Review Board. All participants gave written informed consent prior to participating and before any screening procedures were initiated.

### Pharmacokinetic evaluation

Blood samples of 7 mL were collected in K2EDTA tubes from participants at pre‐dose (0 h) and 0.5, 1.0, 1.5, 2, 4, 6, 12, 24, 48, 72, 96, 120, and 144 h post-oral dose, and at 0, 0.75, 1.25, 1.5, 2, 3, 4, 6, 12, 24, 48, 72, 96, 120, and 144 h post-start of IV dose. Samples were split into 3- and 4-mL tubes, immediately processed to plasma, and stored until analysis at − 20 degrees Celsius for measurement of lorlatinib and at − 70 degrees Celsius for measurement of the metabolite PF-06895751.

Plasma samples were analysed for lorlatinib and PF-06895751 concentrations using validated, sensitive, and specific high-performance liquid chromatographic‐tandem mass spectrometry (LC–MS/MS) methods. The detection range was 0.05–50 ng/mL or 1 to 1000 ng/mL for low and high concentration samples, respectively. Lorlatinib concentrations were measured at Covance Bioanalytical Services (Shanghai, China) and PF-06895751 concentrations were measured at Pfizer Pharmacokinetics, Dynamics and Metabolism (PDM) department (Groton, Connecticut, US). Calibration standard responses were linear over the range of 2.50–2500 ng/mL; using a weighted (l/concentration^2^) linear least squares regression. The between-day assay accuracy, expressed as percent relative error (%RE), for quality control (QC) concentrations, ranged from − 1.6 to 0.8% for the low (7.50 ng/mL), medium (123 ng/mL), and high (1900 ng/mL) QC samples for lorlatinib and ranged from − 8.1 to − 5.8% for the low (7.50 ng/mL), medium (150 ng/mL), and high (1900 ng/mL) QC samples for PF-06895751. Assay precision expressed as the between-day %CV of the mean estimated concentrations of QC samples for lorlatinib was ≤ 6.4% for low, medium, and high concentrations and was ≤ 6.2% for low, medium, and high QC concentrations for PF-06895751. The lower limit of quantification (LLOQ) for lorlatinib and its metabolite was 2.50 ng/mL.

Plasma PK parameters for lorlatinib and its metabolite PF-06895751 were estimated using non-compartmental analysis (NCA) of plasma concentration–time data. PK parameter values were calculated using an internally validated software system (eNCA). Actual sample collection times were used for the PK parameter analysis and samples below the LLOQ were set to 0. Maximum observed plasma concentration, C_max_, and time when C_max_ was reached, T_max_, were determined from the observed values. The area under the curve (AUC) from time 0 to the time of the last quantifiable concentration (AUC_last_) was estimated using the linear/log trapezoidal method. AUC_inf_ was calculated as AUC_last_ + (C_last_/k_el_), where C_last_ was the predicted plasma concentration at the last quantifiable time point estimated from the log-linear regression analysis and k_el_ was the terminal phase rate constant calculated by a linear regression of the log-linear concentration–time curve. PK parameters based on terminal phase estimation were reported only where a well-characterized terminal phase was observed. A well-characterized terminal phase is defined as one with at least three data points, *r*^2^ ≥ 0.9, and AUC_extrap_% ≤ 20. Metabolite-to-parent ratios were calculated after adjustment for molecular weights. Clearance (CL) for the IV dose and CL/F for the oral dose were calculated as dose/AUC_inf_. Volume of distribution during the terminal phase (area) was calculated as IV dose/(k_el_×AUC_inf_).

Since data from a prior study indicated that less than 1% of parent drug is detected in the urine, hepatic clearance was assumed to be the major contributor toward total clearance for lorlatinib [11]. The hepatic extraction ratio was calculated for each participant as the hepatic blood clearance divided by hepatic blood flow. Assuming a hepatic blood flow of 90 L/hr based on the typical blood flow for a 70 kg healthy participant, the hepatic blood clearance for lorlatinib was estimated as the plasma clearance multiplied by the blood-to-plasma partitioning ratio of 0.985.

### Safety evaluation

Safety assessments were performed at screening, during the study, and at study completion, clinic discharge, or early termination. Safety assessments included physical examinations, 12‐lead ECGs, vital signs, and clinical laboratory evaluations. All adverse events (AEs) and concomitant treatments were monitored and recorded throughout the study. AEs were reported using NCI CTCAE terminology.

### Statistical analysis

Summary profiles (mean) of the lorlatinib plasma concentration–time data were plotted by treatment. Nominal PK sampling time was used for summary statistics and summary plots by sampling time. Dose-normalized (dn) natural log-transformed AUC_inf_ (AUC_inf_(dn)) and AUC_-last_(dn) were analysed using a mixed-effect model with sequence, period, and treatment as fixed effects and subject within sequence as a random effect. Estimates of the adjusted geometric mean difference (test–reference, where lorlatinib IV dose was the reference and lorlatinib oral dose was the test) and corresponding 90% confidence interval (CI) were obtained from the model. The adjusted geometric mean difference and 90% CI for the difference were exponentiated to provide estimates of the ratio of adjusted geometric means (test/reference) and 90% CI for the ratios. Absolute bioavailability was expressed as the ratio of dose-normalized adjusted geometric means of AUC_inf_ for lorlatinib oral and IV doses.

### Determination of sample size

The sample size of 12 participants was empirically selected and not based on a statistical power calculation. Participants who failed to complete the study were not to be replaced unless the number of evaluable participants fell below 10. It was determined that a sample size of 12 participants (6/treatment sequence) would provide a 90% CI for the difference between treatments of ± 0.0858 on the natural logarithm scale for AUC, with 80% coverage probability. This estimate was based on four healthy volunteer studies, assuming within-subject standard deviation of 0.10 for natural log-transformed AUC_inf_.

## Results

### Participants

Between 13 Jun 2016 and 2 Sep 2016, a total of 11 participants were randomized to receive one of two study treatment sequences. All participants were male, with a mean age of 37.6 years (24–52 years). The mean body weight was 85 kg (60–110 kg) and the mean BMI was 27.2 kg/m^2^ (19.8–30.5 kg/m^2^). Demographic information is summarized in Table 1.

**Table 1:** Participant demographics

		All participants *N* = 11
Male		11
Age (years)		
18–25		2
26–35		3
36–45		3
46–55		3
Mean (SD)		37.6 (10.3)
Range		24–52
Race		
White	1	
Black	4	
Asian	1	
Other	5	
Weight, kg		
Mean (SD)		85.0 (13.7)
Range		60.0–110.4
BMI, kg/m^2^		
Mean (SD)		27.2 (3.1)
Range		19.8–30.5
Height, cm		
Mean (SD)		176.6 (9.0)
Range		165–191

*BMI* body mass index, *SD* standard deviation

### Pharmacokinetic results

**Fig. 1:**
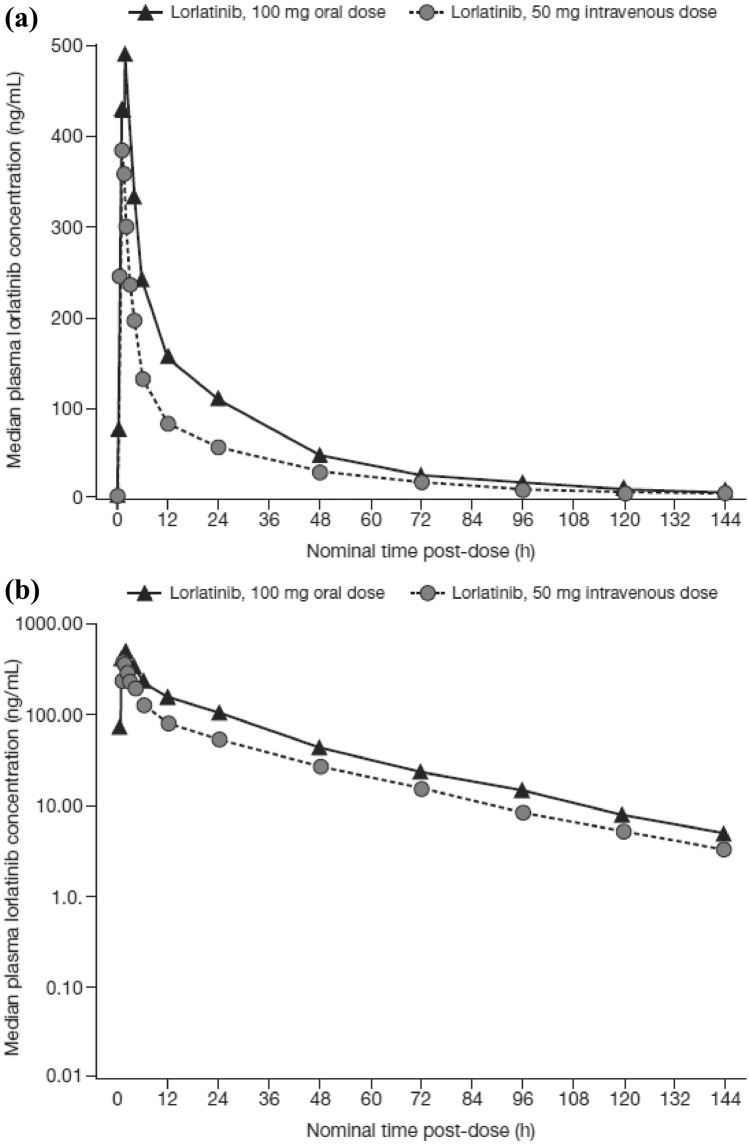
Median plasma lorlatinib concentration–time profiles following lorlatinib oral and IV treatment. Upper and lower panels are linear and semi-logarithmic scales, respectively. Summary statistics were calculated by setting concentration values below the LLOQ to 0. The LLOQ was 2.5 ng/mL. *h* hour(s), *IV* intravenous, *LLOQ* lower limit of quantification

**Fig. 2:**
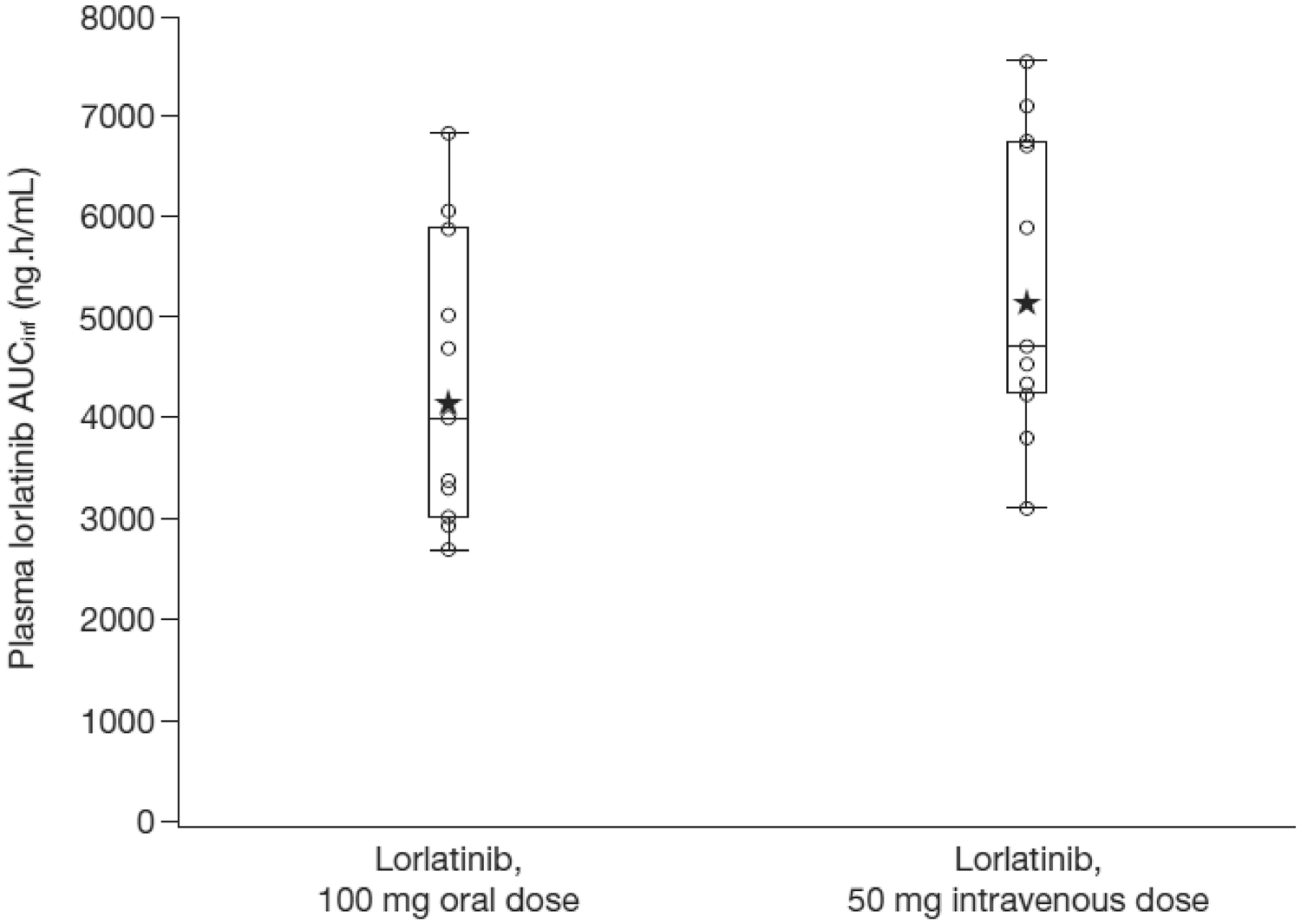
Individual and geometric mean plasma lorlatinib AUC_inf_ values by treatment. Stars represent the geometric mean and open circles represent individual values. Box plot provides median and 25/75% quartiles with whiskers to the last point within 1.5 × interquartile range. The AUC_inf_ for the lorlatinib 100 mg oral dose were normalized to 50 mg of the IV dose. AUC_inf_, AUC_-last_ + (C_last_/k_el_); *h* hour(s), *IV* intravenous

Lorlatinib median plasma concentration–time profiles following oral and IV doses are presented in Fig. 1. None of the participants had any lorlatinib concentration at the start of their second dose. The apparent plasma terminal elimination half-life was similar following IV and oral administration, with mean values of approximately 27.0 and 25.5 h, respectively (Table 2). Inter-subject variability for lorlatinib AUC_inf_, AUC_last_, and C_max_ was similar for both oral administration and IV dosing with %CV values in the range of 30–38% (Table 2). Boxplots with individual and geometric mean AUC_inf_(dn) are plotted in Fig. 2. The geometric mean (geometric coefficient of variation, %) hepatic extraction ratio was estimated as 0.118 (30%). A full summary of plasma PK parameter values following oral and IV dosing is provided in Table 2. Total plasma exposures of lorlatinib following oral administration relative to IV infusion, normalized for dose, were 80.78% (90% CI: 75.73–86.16%) and a statistical comparison for the adjusted geometric mean absolute oral bioavailability for lorlatinib is presented in Table 3.

**Table 2:** Summary of lorlatinib plasma pharmacokinetic parameter values by treatment

Parameter, units	Lorlatinib 100 mg oral tablets *n* = 11 Geometric mean (CV%)	Lorlatinib 50 mg IV *n* = 11 Geometric mean (CV%)
AUC_inf_, ng.h/mL	8289 (34)	5148 (30)
AUC_last_, ng.h/mL	8109 (33)	4986 (30)
C_max_, h	501.3 (38)	392.5 (34)
T_max_, h	1.50 (1.00–4.02)	1.28 (1.27–1.50)
t½, h	25.54 ± 3.75	27.02 ± 5.05
CL/F, L/h	12.05 (34)	NA
V_z_/F, L	440.2 (28)	NA
CL, L/h	NA	9.713 (30)
Vss, L	NA	304.8 (28)
AUC_inf_, ng.h/mL (dn)	4143 (34)	5148 (30)
AUC_last_, ng.h/mL (dn)	4058 (33)	4986 (30)
C_max_, ng/mL (dn)	250.9 (38)	392.5 (34)
E_h_	0.118 (30)	NA

Geometric means (geometric %CV) are provided for all parameters except median (range) is provided for T_max_ and arithmetic mean (± SD) for t½. Values were normalized to 50 mg IV dose. n, number of subjects in the treatment group and contributing to the summaries for all PK parameters

AUC_last_, area under the curve (AUC) from time 0 to the time of the last quantifiable concentration; AUC_inf_, AUC_-last_ + (C_last_/k_el_); C_max_, maximum observed plasma concentration; CL, clearance; CL/F, apparent clearance; CV%, percent coefficient of variation; *Eh* hepatic extraction, *dn* dose normalized, *h* hour(s), *IV* intravenous, *NA* not applicable, *SD* standard deviation, *T*_max_ time for C_max_ to be reached, *t*½ plasma terminal half-life, *Vz/F* volume of distribution

**Table 3:** Statistical summary of treatment comparison for lorlatinib pharmacokinetic parameters

	Adjusted geometric means			
Parameter, units	Test Lorlatinib 100 mg oral tablets	Reference Lorlatinib 50 mg IV	Ratio (test/reference) of adjusted means	90% CI for ratio
AUC_inf_, ng.h/mL (dn)	4191	5189	80.78	(75.73–86.16)
AUC_last_, ng.h/mL (dn)	4106	5028	81.65	(76.56–87.08)

Values had been back-transformed from the log scale. The mixed-effects model included sequence, period, and treatment as fixed effects and participant within sequence as a random effect. The ratios (and 90% CIs) were expressed as percentages. Values normalized to 50 mg IV dose

AUC_last_, area under the curve (AUC) from time 0 to the time of the last quantifiable concentration; AUC_inf_, AUC_-last_ + (C_last_/k_el_); *CI* confidence interval, *dn* dose normalized, *h* hour(s), *IV* intravenous

**Fig. 3:**
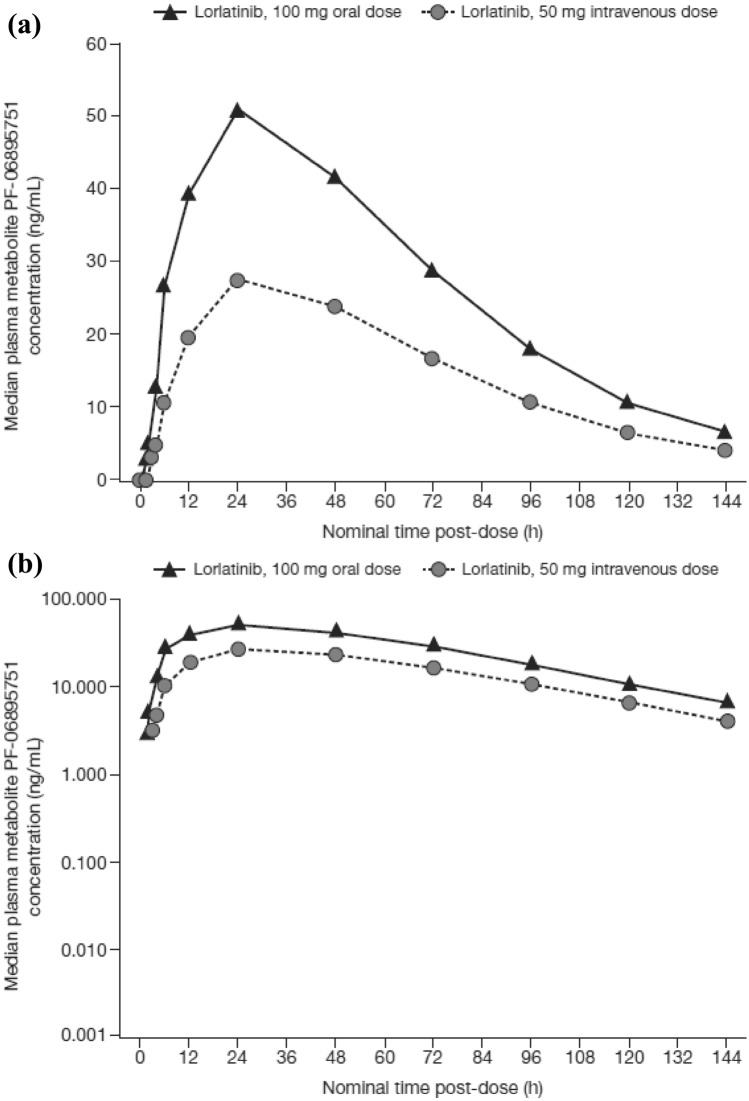
Median plasma metabolite PF-06895751 concentration–time profiles following lorlatinib oral and IV treatment. Upper and lower panels are linear and semi-logarithmic scales, respectively. Summary statistics had been calculated by setting concentration values below LLOQ to 0. The LLOQ was 2.5 ng/mL. *h* hour(s), *IV* intravenous, *LLOQ* lower limit of quantification

Median plasma concentration–time profiles for metabolite PF-06895751 following oral and IV doses are presented in Fig. 3. A summary of PF-06895751 plasma PK parameter values following oral and IV dosing is provided in Table 5.

### Safety evaluation

All 11 participants who completed the study were evaluated for safety. No deaths, serious AEs, severe AEs, permanent discontinuations, temporary discontinuations, or dose reductions due to AEs were reported. There was an equal number of participants who reported AEs in each treatment, with more AEs reported following the lorlatinib 100 mg oral treatment (12 AEs in 4 participants) compared to the lorlatinib 50 mg IV treatment (8 AEs in 4 participants) (Table 4). Only two AEs (transaminase increased) reported in one participant were considered moderate in severity and clinically significant. The most common treatment-emergent AE (TEAE) was headache, which was reported by two participants with each treatment regimen (Table 4). The majority of the TEAEs (6 out of 8 for IV treatment and 9 out of 12 for oral treatment) were considered treatment-related AEs (TRAEs) by the Investigator. All TEAEs were resolved by the end of the study without significant treatment intervention.

**Table 4:** Incidence of adverse events by treatment

Number of participants with AEs by system organ class and MedDRA preferred term	Lorlatinib 100 mg oral tablets *n* = 11 All causality (TRAE)	Lorlatinib 50 mg IV *n* = 11 All causality (TRAE)
Eye disorders	1 (1)	0
Photophobia	1 (1)	0
Gastrointestinal disorders	2 (0)	0
Abdominal discomfort	1 (0)	0
Nausea	1 (0)	0
General disorders and administration side conditions	1 (0)	0
Influenza-like illness	1 (0)	0
Injury, poisoning and procedural complications	0	1 (0)
Contusion	0	1 (0)
Investigations	1 (1)	1 (1)
Transaminase increased	1 (1)	1 (1)
Nervous system disorders	3 (3)	2 (2)
Dizziness	1 (1)	1 (1)
Headache	2 (2)	2 (2)
Mental impairment	1 (1)	0
Paraesthesia	1 (1)	1 (1)
Somnolence	1 (1)	0
Visual field defect	0	1 (1)
Psychiatric disorders	0	1 (0)
Nervousness	0	1 (0)
Renal and urinary disorders	1 (1)	0
Dysuria	1 (1)	0
TEAEs (TRAEs)	12 (9)	8 (6)

Participants were counted only once per treatment in each row. All data collected since the first dose of study drug were included

*AE* adverse event, MedDRA medical dictionary for regulatory activities (version 19.0), *TEAE* treatment-emergent adverse events, *TRAEs* treatment-related adverse events

## Discussion

In this study, clinical data from 11 healthy adults determined the absolute oral bioavailability of lorlatinib after a 100 mg single oral dose relative to a 50 mg IV dose. It was estimated to be 81%, indicating that the lorlatinib oral dose was well absorbed and had a low first-pass effect. These results are supported by in vitro permeability studies that demonstrated lorlatinib is highly permeable [13]. The variability estimates for lorlatinib plasma exposure following IV dose versus oral dose were similar (CV% values in the range of 30–38%), indicating that oral absorption of lorlatinib is not a major source of overall inter-subject variability in lorlatinib disposition. That is, variability in metabolism is likely the predominant contributor to overall PK variability for lorlatinib and any further efforts to develop alternate formulations to improve absorption would not be of benefit.

Consistent with these findings, lorlatinib is an inducer of CYP3A, via human pregnane X receptor activation, as well as an inhibitor of CYP3A with the net effect in vivo being induction. Lorlatinib also induces CYP2B6 and activates the human constitutive androstane receptor [8]. Previous studies have found that lorlatinib plasma exposure can be affected by inducers or inhibitors of CYP3A. For example, plasma exposure was significantly reduced when co-administered with the strong CYP3A inducer rifampicin (AUC_inf_ reduction of 85%) and the moderate CYP3A inducer modafinil (AUC_inf_ reduction of 23%) [9, 10]. Similarly in a study with the strong CYP3A inhibitor, itraconazole, plasma exposure of lorlatinib was increased (AUC_inf_ increase of 42%) [8].

Prior knowledge of the extraction ratio allows the drug development scientist to better anticipate changes in pharmacokinetics of the drug during future drug interaction studies. The lorlatinib hepatic extraction ratio was low, with approximately 12% of lorlatinib metabolized during a single pass through the liver. Hence, lorlatinib can be considered a ‘low’ clearance drug. A low extraction ratio drug (hepatic extraction < 30%) will usually demonstrate a change in the terminal plasma half when co-administered with another drug that is a metabolic inhibitor or inducer. A high extraction ratio drug (> 70% extraction) on the other hand will not show any change in terminal plasma half-life when given with a metabolic inhibitor or inducer. Additionally, high extraction drugs are usually associated with higher inter-subject variability in pharmacokinetics. In the previous drug interaction studies, lorlatinib mean half-life increased from 23 to 30 h with the inhibitor itraconazole and lorlatinib mean half-life decreased from 29 to 18 h with the inducer rifampin, which further validates our assessment of lorlatinib being a low extraction drug.

This study was conducted early in clinical development of lorlatinib, and hence did not use the final commercial formulation. Nonetheless, appropriate bioequivalence studies were conducted to link the oral formulation used in the study with the commercial formulation. Further, although this study used 4 × 25 mg tablets for the 100 mg oral dose, the results are considered interchangeable with the 100 mg tablet since in vitro results showed that the solubility of lorlatinib in aqueous media decreases over the range of pH 2.55–8.02 from 32.38 mg/mL to 0.17 mg/mL.

Of the 14 participants admitted to the study, 3 did not meet study entry criteria and were disqualified prior to any study dosing. The sample size of 12 participants was empirical, and since it was pre-determined that a decrease in up to 2 participants would not decrease the reliability of the study results, it was considered appropriate that only 11 participants completed the study.

## Conclusion

Orally administered lorlatinib is well absorbed with low first-pass effect (12%) and estimated absolute oral bioavailability of 81% (90% CI 75.7, 86.2%). Most treatment-emergent AEs were mild in severity, indicating that lorlatinib is generally well-tolerated when administered as a 50 mg IV dose or as a 100 mg single oral dose in healthy participants.

## Data Availability

Upon request, and subject to certain criteria, conditions, and exceptions (see https://www.pfizer.com/science/clinical-trials/trial-data-and-results for more information), Pfizer will provide access to individual de-identified participant data from Pfizer-sponsored global interventional clinical studies conducted for medicines, vaccines, and medical devices (1) for indications that have been approved in the US and/or EU or (2) in programmes that have been terminated (i.e., development for all indications has been discontinued). Pfizer will also consider requests for the protocol, data dictionary, and statistical analysis plan. Data may be requested from Pfizer trials 24 months after study completion. The de-identified participant data will be made available to researchers whose proposals meet the research criteria and other conditions, and for which an exception does not apply, via a secure portal. To gain access, data requestors must enter into a data access agreement with Pfizer Inc.

## Appendix

See Appendix Fig 3 and Table 5.

**Table 5:** Summary of plasma metabolite PF-06895751 pharmacokinetic parameter values by treatment

Parameter, units	Lorlatinib 100 mg oral tablets *N* = 11, *n* = 10 Geometric mean (CV%)	Lorlatinib 50 mg IV *N* = 11, *n* = 11 Geometric mean (CV%)
AUC_inf_, ng.h/mL	4399 (23)	2310 (21)
AUC_last_, ng.h/mL	3870 (27)	2091 (23)
C_max_, ng/mL	47.01 (33)	26.74 (24)
T_max_, h	24.0 (12.1–48.0)	24.0 (24.0–48.1)
t½, h	32.84 ± 6.13	34.45 ± 6.31
MRAUC_inf_	1.187 (38)	0.9903 (40)
MRAUC_last_	1.053 (43)	0.9253 (43)
MRC_max_	0.2070 (34)	0.1504 (41)

Geometric means are provided for all parameters except median (range) is provided for T_max_ and arithmetic mean (± SD) for t½. *N* number of subjects in the treatment group and contributing to the summaries, *n* number of subjects with reportable AUC_inf_, t½, and MRAUC_inf_ values. MR parameters were calculated after correction for molecular weight differences (convert from ng to nmol),

*AUC*_*last*_ area under the curve (AUC) from time 0 to the time of the last quantifiable concentration, AUC_inf_, AUC_-last_ + (C_last_/k_el_); *C*_*max*_ maximum observed plasma concentration, *CV%* percent coefficient of variation, *dn* dose normalized, *h* hour(s), *IV* intravenous, *MR* metabolite-to-parent ratio, *SD* standard deviation, *T*_*max*_ time for C_max_ to be reached, *t½*, terminal plasma half-life

## References

[1] Zou HY, Friboulet L, Kodack DP, Engstrom LD, Li Q, West M (2015). PF-06463922, an ALK/ROS1 inhibitor, overcomes resistance to first and second generation ALK inhibitors in preclinical models. Cancer Cell.

[2] Shaw AT, Solomon BJ, Chiari R, Riely GJ, Besse B, Soo RA (2019). Lorlatinib in advanced *ROS1*-positive non-small-cell lung cancer: a multicentre, open-label, single-arm, phase 1–2 trial. Lancet Oncol.

[3] Solomon BJ, Besse B, Bauer TM, Felip E, Soo RA, Camidge DR (2018). Lorlatinib in patients with *ALK*-positive non-small-cell lung cancer: results from a global phase 2 study. Lancet Oncol.

[4] Syed YY (2019). Lorlatinib: first global approval. Drugs.

[5] Shaw AT, Bauer TM, de Marinis F, Felip E, Goto Y, Liu G, Mazieres J, Kim DW, Mok T, Polli A, Thurm H, Calella AM, Peltz G, Solomon BJ, CROWN Trial Investigators (2020). First-line Lorlatinib or Crizotinib in advanced ALK-positive lung cancer. N Engl J Med.

[6] Pfizer Inc. LORBRENA^®^ (lorlatinib): Prescribing Information. 2020. Available at http://labeling.pfizer.com/ShowLabeling.aspx?id=11140 (accessed Jul 13, 2020).

[7] Chen J, O’Gormon MT, James LP, Klamerus KJ, Mugundu G, Pithavala YK (2021). Pharmacokinetics of Lorlatinib after single and multiple dosing in patients with anaplastic lymphoma kinase (ALK)-positive non-small cell lung cancer: results from a global phase I/II study. Clin Pharmacokinet.

[8] Patel M, Chen J, McGrory S, O'Gorman M, Nepal S, Ginman K (2020). The effect of itraconazole on the pharmacokinetics of lorlatinib: results of a phase I, open-label, crossover study in healthy participants. Invest New Drugs.

[9] Chen J, Xu H, Pawlak S, James LP, Peltz G, Lee K (2020). The Effect of Rifampin on the pharmacokinetics and safety of Lorlatinib: results of a phase one, open-label, crossover study in healthy participants. Adv Ther.

[10] Li J, Pithavala YK, Gong J, LaBadie RR, Mfopou JK, Chen J (2021). The effect of Modafinil on the safety and pharmacokinetics of Lorlatinib: a phase I study in healthy participants. Clin Pharmacokinet.

[11] Stypinski D, Fostvedt L, Lam JL, Vaz A, Johnson TR, Boerma JS (2020). Metabolism, excretion, and pharmacokinetics of Lorlatinib (PF-06463922) and evaluation of the impact of radiolabel position and other factors on comparability of data across 2 ADME studies. J Clin Pharmacol.

[12] Shaw AT, Felip E, Bauer TM, Besse B, Navarro A, Postel-Vinay S (2017). Lorlatinib in non-small-cell lung cancer with *ALK* or *ROS1* rearrangement: an international, multicentre, open-label, single-arm first-in-man phase 1 trial. Lancet Oncol.

[13] Johnson TW, Richardson PF, Bailey S, Brooun A, Burke BJ, Collins MR, Cui JJ, Deal JG, Deng YL, Dinh D, Engstrom LD, He M, Hoffman J, Hoffman RL, Huang Q, Kania RS, Kath JC, Lam H, Lam JL, Le PT, Lingardo L, Liu W, McTigue M, Palmer CL, Sach NW, Smeal T, Smith GL, Stewart AE, Timofeevski S, Zhu H, Zhu J, Zou HY, Edwards MP (2014). Discovery of (10R)-7-amino-12-fluoro-2,10,16-trimethyl-15-oxo-10,15,16,17-tetrahydro-2H-8,4-(metheno)pyrazolo[4,3-h][2,5,11]-benzoxadiazacyclotetradecine-3-carbonitrile (PF-06463922), a macrocyclic inhibitor of anaplastic lymphoma kinase (ALK) and c-ros oncogene 1 (ROS1) with preclinical brain exposure and broad-spectrum potency against ALK-resistant mutations. J Med Chem.

