# Correction to: Evaluation of the absolute oral bioavailability of the anaplastic lymphoma kinase/c-ROS oncogene 1 kinase inhibitor lorlatinib in healthy participants

**DOI:** 10.1007/s00280-022-04421-7

**Published:** 2022-03-18

**Authors:** Jennifer E. Hibma, Melissa O’Gorman, Sunil Nepal, Sylvester Pawlak, Katherine Ginman, Yazdi K. Pithavala

**Affiliations:** 1 Pfizer (Clinical Pharmacology Oncology), La Jolla, 10555 Science Center Drive, CB10-2727, San Diego, CA 92121 USA; 2 Pfizer (Pharmacometrics Oncology), Groton, CT USA; 3 Pfizer (Statistics Oncology), Groton, CT USA; 4 Pfizer (Clinical Development and Operations), New Haven, CT USA; 5 Pfizer (Clinical Development and Operations), South Lyon, MI USA; 6 Independent Statistical Consultant, Schwenksville, PA USA

## Correction to: Cancer Chemotherapy and Pharmacology (2022) 89:71–81 10.1007/s00280-021-04368-1

The article “Evaluation of the absolute oral bioavailability of the anaplastic lymphoma kinase/c-ROS oncogene 1 kinase inhibitor lorlatinib in healthy participants”, written by Jennifer E. Hibma · Melissa O'Gorman · Sunil Nepal · Sylvester Pawlak · Katherine Ginman and Yazdi K. Pithavala was originally published Online First without Open Access. After publication in volume 89, issue 1, page 71–81 the author decided to opt for Open Choice and to make the article an Open Access publication. Therefore, the copyright of the article has been changed to © The Author(s) 2022 and the article is forthwith distributed under the terms of the Creative Commons Attribution 4.0 International License (https://creativecommons.org/licenses/by/4.0/), which permits use, sharing, adaptation, distribution and reproduction in any medium or format, as long as you give appropriate credit to the original author(s) and the source, provide a link to the Creative Commons licence, and indicate if changes were made.

The original article has been corrected.

